# Rituximab induced acute thrombocytopenia in a patient with systemic lupus erythematosus: a case report

**DOI:** 10.1186/s13256-021-02950-y

**Published:** 2021-07-08

**Authors:** Jevon Yudhishdran, Jeyalakshmy Sivakumar, Mitrakrishnan Rayno Navinan, Sareesh Bandapatti

**Affiliations:** 1 District General Hospital, Killinochi, Sri Lanka; 2 National Hospital of Sri Lanka, Colombo, Sri Lanka; 3 Apollo Hospital, Hydrabad, India

**Keywords:** Systemic lupus erythematosus, Rituximab, Thrombocytopenia, Bleeding, Isolated, Acute

## Abstract

**Background:**

Rituximab is a novel chimeric monoclonal antibody that has established itself as a potent therapeutic option for autoimmune medical conditions, including systemic lupus erythematosus, owing to its mechanism of action targeting CD20 cells. Rituximab is also known to cause a spectrum of side effects including hematological abnormalities. Acute isolated thrombocytopenia following rituximab is an uncommon occurrence and, when seen, occurs in the presence of underlying hematological malignancies. Its occurrence in autoimmune diseases is rare. Despite this, acute isolated thrombocytopenia in the backdrop of systemic lupus erythematosus is undocumented.

**Case presentation:**

A young 36-year-old South Asian female with systemic lupus erythematosus with class IV lupus nephritis poorly responding to standard therapy was initiated on rituximab. Ten days later, she presented with mucocutaneous bleeding and ecchymotic skin lesions. Isolated severe thrombocytopenia was noted with a platelet count of 5 × 10^9^/L (150–450). Anticipating life-threatening bleeding, she was given intravenous immunoglobulin, methyl prednisolone, and platelet transfusion considering a spectrum of initial differential diagnosis. Rituximab was also withheld. Though extensively investigated, most investigations were negative. A platelet destructive process was suspected as bone marrow biopsy showed adequate megakaryocytes. Weighing the risk versus benefit, following recovery, she was reinitiated on rituximab. Within 4 days, she presented again with similar symptoms and severe isolated thrombocytopenia was noted. Rituximab-induced acute thrombocytopenia was considered the working clinical diagnosis.

**Case discussion and conclusion:**

Rituximab can cause a spectrum of hematological abnormalities, including isolated acute thrombocytopenia. Its occurrence in autoimmune conditions is rare, and its manifestation in systemic lupus erythematosus is undocumented. Its exact etiology is still disputed. Usually considered benign, the platelet numbers tend to show improvement with cessation of therapy. However, in the presence of mucocutaneous bleeding in our patient, we took an aggressive approach to management. Though evidence for corrective therapy is anecdotal, it could be justified on the basis of averting potential catastrophic hemorrhagic manifestations. The spectrum of autoimmune disease that potentially predisposes rituximab to cause thrombocytopenia should be extended to include systemic lupus erythematosus.

## Introduction

Rituximab is an intravenous chimeric monoclonal antibody [1] developed from deoxyribonucleic acid technology using human and mice genes that act via CD 20 receptors. Since it has immune-modulatory action with biologic activity, its spectrum of use has increased to include a number of autoimmune disorders [2]. The multifunctionary role of B cells in systemic lupus erythematosus (SLE) and its depletion by targeting CD20 by using rituximab [3] has resulted in its off-label use for conditions such as SLE , and has been acknowledged to produce good clinical efficacy [4] and is specially indicated for moderate to severe forms of SLE that are refractory to therapy [5]. The use of rituximab also comes with a caveat of potentially extensive list of possible side effects, and can afflict the hematological and lymphatic system. The observed spectrum of recognized hematological abnormalities affects all cell lines and can result in in anemia, leukopenia (neutropenia and lymphopenia), and even thrombocytopenia [6]. Delayed pancytopenia following rituximab is recognized, but early isolated thrombocytopenia following its use is considered uncommon [7]. We present a case of isolated early thrombocytopenia occurring following rituximab use presenting with acute mucocutaneous hemorrhagic manifestations.

## Case presentation

A 36-year-old South Asian female was on surveillance and clinic follow-up for SLE complicated with class IV lupus nephritis and hypertension since 2012. She had no other significant medical, surgical, allergic, or family history of significance. She was on oral prednisolone 10 mg once daily and mycophenolate mofetil (MMF) 1 g twice daily as maintenance immunosuppressive treatment along with oral enalapril 10 mg once at night, oral diltiazem 30 mg three times a day, oral hydroxychloroquine 100 mg once daily, oral alendronic acid 35 mg once a week, and oral omeprazole 20 mg once a day. Throughout her follow-up, she maintained good hemodynamic parameters, being normotensive and with heart rates within reference ranges. Her whole blood analysis demonstrated a white cell count of 6.9 × 10^9^/L (4–11 × 10^9^/L), hemoglobin of 11.3 g/dL (11–15 g/dL), and platelet count of 222 × 10^9^/L (150–450 × 10^9^/L). Her remaining blood tests including renal function and inflammatory markers, as well as urinalysis, were normal (Table 1). She was in clinical remission. However, in view of possible conception following a multidisciplinary discussion, her MMF was converted to oral azathioprine 50 mg twice daily based on both safety profile and feasibility of treatment. Her hydroxychloroquine was continued. Though therapy was changed in expectation, she failed to conceive, and subsequently she showed clinical regression with persistent proteinuria. In view of that, she was recommenced on MMF and on a tapering-down regimen of oral prednisolone to treat the relapse. Despite appropriate dose and compliance, the proteinuria persisted, and a repeat renal biopsy was done to exclude class shift. Renal biopsy revealed a single crescent was present with 17 glomeruli visualized and corresponded to a responding class IV nephritis with acute index of 6/24 and a chronic index of 1/12. In light of this and failure to respond to MMF and steroids, a clinical decision was taken to step up therapy and try rituximab in addition. The patient was given the first dose of intravenous rituximab at 375 mg/m^2^. However, 10 days after the rituximab therapy, she presented to the emergency department with complaints of spontaneous bleeding from the mouth and nose of 1-day duration along with new-onset patchy ecchymotic lesions throughout the body.

**Table 1:** Baseline laboratory parameters

Investigation and reference range	Value
Full blood count	
White blood cells, × 10^9^/L (4–11)	6.9
Hemoglobin, g/dL (11–15)	11.3
Platelet count, × 10^9^/L (150–450)	222
Renal function	
Serum sodium, mmol/L (135–145)	136
Serum potassium, mmol/L (3.5–5.1)	4.3
Serum creatinine, μmol/L (44–97)	52
Blood urea, mmol/L (2.5–7.1)	4.3
Inflammatory markers	
Erythrocyte sedimentation rate, mm/hour (< 20)	20
C-Reactive protein, mg/dl (< 5)	3.2
Urinalysis	
Protein	Trace
White cells, /high-power field (HPF) (2–5)	1–2
Red cells, /HPF (< 2)	Nil

**Fig. 1:**
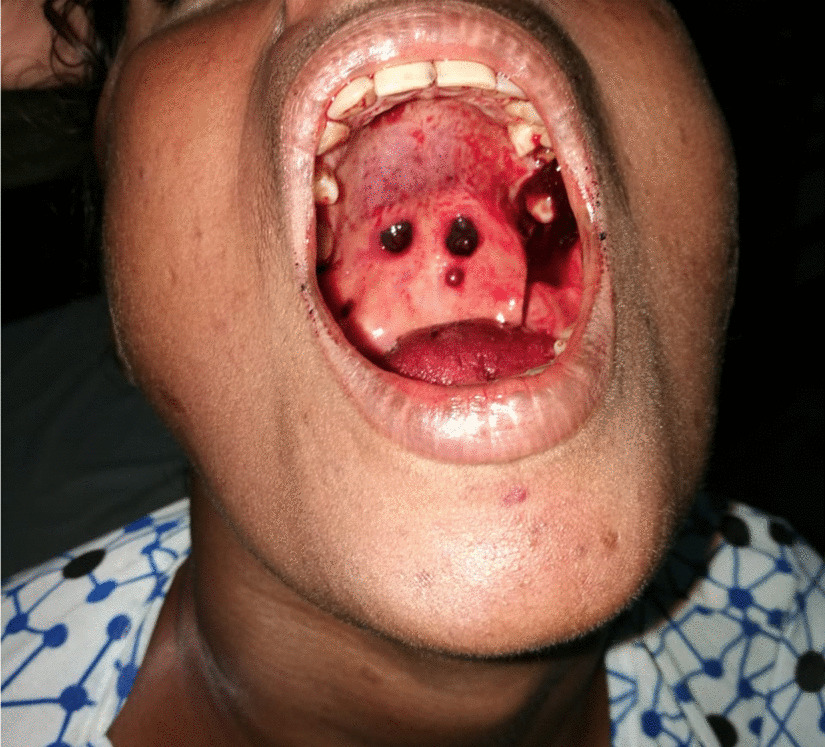
Picture demonstrating multiple blood filled blisters in the hard part of the palate with petechiae in the soft palate with bleeding from the left upper molar

Other than the stated symptoms, she had no additional complaints. Further questioning failed to reveal other sites of bleeding. Clinical examination revealed blood oozing from the gums and two blood blisters on the hard palate (Fig 1); blood was noted on the alar nasi without any crusting. She was also noted to have multiple ecchymotic patches throughout her body. The tip of the spleen was palpable, being soft and nontender on examination of the abdomen. Remainder of the systemic examination was normal. Initial evaluation with whole blood analysis revealed severe thrombocytopenia with a platelet count of only 5 × 10^9^/L (150–450 × 10^9^/L) with hemoglobin 10.2 g/dL (11–15 g/dL), and white cell count of 6.8 × 10^9^/L (4–11 × 10^9^). The blood picture during admission showed severe thrombocytopenia without any abnormal cells, and other cell lines were normal. Serum creatinine was 98 μmol/L, serum albumin was 4.03 g/dl, international normalized ratio was normal at 0.92 (< 1.0), and C-reactive protein was only 0.6 (< 5.0 mg/dl). Coombs direct agglutination test was negative.

**Fig. 2:**
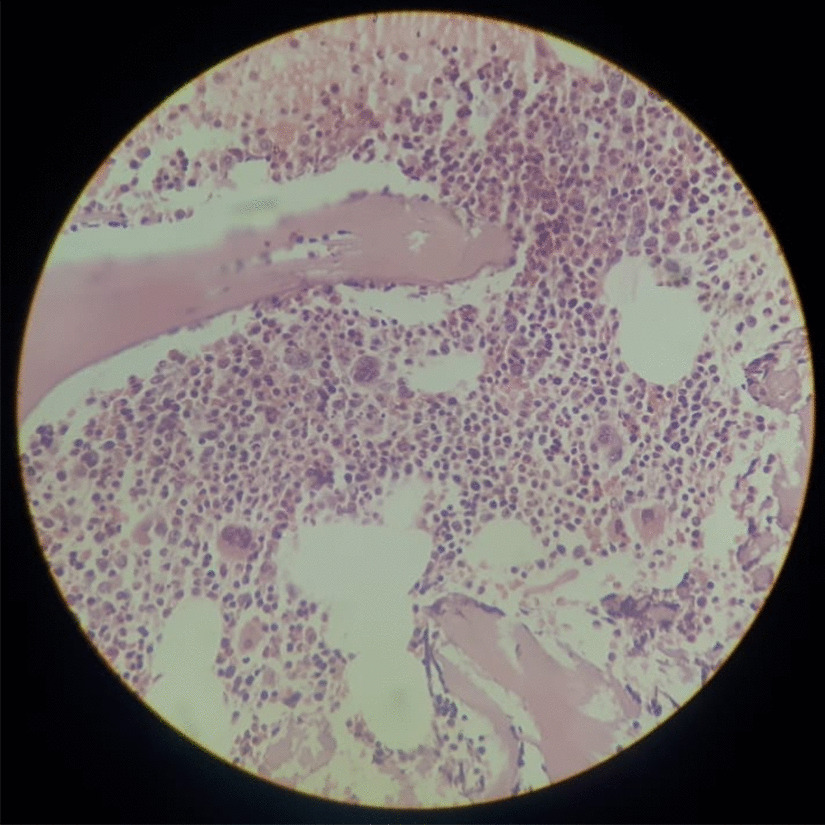
Bone marrow trephine biopsy demonstrating morphologically normal trilinear hematopoietic elements. Adequate number of megakaryocytes are seen in the absence of fibrosis, granuloma formation, hematological or nonhematological malignant infiltrates

Due to the severe thrombocytopenia considering the high risk of intracerebral hemorrhage, she was given platelet transfusion (five pooled packs) and was empirically given 5 days of intravenous immunoglobulin (IG) at 0.4 g/kg body weight along with intravenous methylprednisolone 1 g daily for 3 days suspecting immune thrombocytopenic purpura (ITP). On the subsequent day, the platelet counts remained at 7 × 10^9^/L (150–450 × 10^9^/L) (Table 2), and repeat whole blood analysis with manual counting done 6 hours later further confirmed a platelet destructive process. Given her background of SLE, a bone marrow biopsy was performed, which demonstrated adequate number of megakaryocytes in the bone marrow compatible with a platelet destructive process [Fig 2]. She was kept under observation with daily whole blood analysis. This revealed gradually rising platelet counts, and 10 days following admission when her platelet count exceeded 100 × 10^9^/L, she was discharged from hospital care. Oral prednisolone dose was tailed off as per guidelines and brought down to her maintenance dose for nephropathy of 7.5 mg once daily, and the patient maintained platelet counts above 200 × 10^9^/L (150–450 × 10^9^/L) on follow-up 1 month later. Although rituximab-induced thrombocytopenia was entertained as a differential, the platelet destructive process, lack of previous reported similar cases in SLE patients, and the necessity of treating the class IV nephritis was weighed and a clinical decision was taken to resume rituximab treatment considering it was a less likely etiology. Her bloods done before infusion showed a platelet count of 283 × 10^9^/L (150–450 × 10^9^/L). She received her second dose of rituximab 375 mg 3 months after her initial presentation to the Accident and Emergency (A & E). Four days later, she again presented to the emergency department with recurrence of oral bleeding. Complete blood count showed severe thrombocytopenia with a platelet count of 10 × 10^9^/L (150–450 × 10^9^/L). The rest of her blood work was within normal limits. In view of her previous admission and rapid response to treatment, she was started on intravenous methylprednisolone 1 g daily for 3 days to be followed by oral prednisolone, along with a 5-day course of intravenous IG at 0.4 g/kg body weight. By day 3, the platelet count started to rise and reached 110 × 10^9^/L (150–450 × 10^9^/L) on day 5, and the patient was discharged from ward and reviewed in clinic a week later (platelet trends presented in Table 2). Whole blood analysis revealed platelet counts within normal reference range, and she was given a plan for rapid taper on a weekly interval to bring the prednisolone dose down to her maintenance value of 7.5 mg/day, and MMF was reinitiated. Given the association between rituximab-induced thrombocytopenia and lymphoma and the patient’s past history of SLE and immune-modulatory drug use, a contrast-enhanced CT scan of the chest, abdomen, and pelvis was performed and revealed a normal-sized spleen and no significant abnormality.

**Table 2:** Pattern of platelet count derangement before and after rituximab administration

	Baseline	First admission (10 days after first cycle of rituximab)					Baseline	Second admission (4 days after second cycle of rituximab)		Baseline
	Before initiation of rituximab	Day 1	Day 2	Day 3	Day 5	Day 10	After recovery and before second cycle of rituximab	Day 1	Day 5	After recovery from second cycle of rituximab and on clinic review
Hb (g/dL)	11.3	11.8	10.0	10.0	10.7	11.1	11.7	10.2	10.7	12.1
WBC (× 10^9^/L)	6.9	8.3	8.6	9.1	8.6	8.7	6.6	6.8	9.2	7.2
Platelet (× 10^9^/L)	222	5	7	21	72	100	283	10	110	261

Hb, hemoglobin; WBC, white blood cells

The patient was planned for monthly follow-ups with a review of counts and monitoring of peripheral lymph nodes and ultrasound examinations to check for paraaortic lymph nodes. Follow-up for 3 months showed no lymphadenopathy, and cell counts remained within normal margins. The final working diagnosis was rituximab-induced isolated thrombocytopenia with mucocutaneous hemorrhagic manifestations.

## Discussion

Isolated thrombocytopenia following rituximab use is observed but usually on the background of hematological malignancies, such as non-Hodgkin’s lymphoma [6] and leukemias (hairy cell leukemia, prolymphocytic leukemia) [8]. Overall, the presence of oncological pathology, bone marrow infiltration, splenomegaly, underlying thrombocytopenia, and high platelet distribution width have been recognized as preexisting risk factors as opposed to autoimmune disease per se [7, 9] when thrombocytopenia is seen following rituximab use. The occurrence of thrombocytopenia in SLE following rituximab is undocumented.

The exact mechanism is still debated, and multiple etiopathogeneses are considered, including consumptive coagulopathy, immune pathway mediated platelet degradation, CD 20 based antigen–antibody reaction, and cell lysis and intravascular fibrinolysis [7, 8], but these hypothesis are based mainly on when thrombocytopenia was observed in patients with hematological malignancy. Thus, the basis of above-mentioned theories being potentially applicable in this case is debatable.

Additionally, thrombocytopenia in SLE can have numerous etiologies including, for example, antibodies (anti-glyco-protein IIb/IIIa and anti-thrombopoietin receptor) against platelets, thrombotic thrombocytopenic purpura, peripheral consumption, bone marrow disease including aplastic anemia and myelofibrosis [10–12]. But when these underlying pathologies are present, usually thrombocytopenia is seen on initial assessment of most patients with SLE [10]. Prior to initiation, our patient had normal platelet counts, and clinical examination was also normal. Following the event, bone marrow assessment and computed tomography imaging failed to reveal any obvious underlying sinister pathology that could account for or associate with a low platelet count, and instead the bone marrow showed a platelet destructive process. Additionally, the return of platelet counts to normal levels and recurrence of the same on reinitiation suggest a causal relationship.

The clinical pattern of thrombocytopenia following rituximab administration is usually considered transient with resolution and typically follows a benign course and usually does not result in bleeding manifestations [13, 14]. The general consensus regarding thrombocytopenia and hemorrhagic manifestation is that numbers alone do not dictate bleeding; instead, the impairment of its function is what plays a major role and, hence, commonly, minor and not life-threatening bleeding is observed [15]. But, in advanced thrombocytopenia grade 4, especially when numbers decrease below 10 × 10^9^/L, life-threatening bleeds become a possibility [16]. Thus to err on the side of caution, platelet transfusion has been given as a precautionary measure even in rituximab-induced thrombocytopenia when the situation was warranted [13]. Our patient demonstrated a typical clinical time line, but contrary to common observation, our clinical vignette demonstrated mucocutaneous bleeding manifestations. Due to the severity of the level of thrombocytopenia, a clinical consensus was taken, and the patient was transfused platelets and simultaneously initiated on alternate oral and intravenous immunomodulatory therapy to correct and expedite normalization of platelets in the hope that the underlying etiology was immune mediated.

The general recommendation is the careful observation of whole blood levels following administration of rituximab and to expectantly monitor for potential drops in platelet count that could signify this adverse reaction. SLE, being a relatively unknown association with rituximab-induced thrombocytopenia, did not raise our clinical suspicion initially to warrant it as a definitive diagnosis, but the clear reoccurrence following reinitiation confirmed this was rituximab-induced thrombocytopenia in the background of SLE. The anecdotal evidence for treatment in such situations and the potential catastrophic implications would make any clinician question the role of vigilance versus active intervention. We chose aggressive therapy to err on the side of caution in the absence of clear guidance and consensus in such a rare clinical scenario. Whether treatment with immunosuppressive therapy had any impact or whether self-resolution occurred is debatable.

## Conclusion

Rituximab-induced thrombocytopenia is an infrequent occurrence and, when seen, is usually in the background of hematological malignancies. Its occurrence in autoimmune disease is rare. Additionally, rituximab causing thrombocytopenia in SLE is not documented in literature. Though generally thought to be benign, the thrombocytopenia can result in hemorrhagic manifestations. Guidance is required on how to manage such situations as severe thrombocytopenia can cause catastrophic events, and clear consensus is necessary to better guide clinical decisions.

## Data Availability

All the data used and or analyzed during case report development have been included in the case presentation.

## Abbreviations

SLE: Systemic lupus erythematosus
MMF: Mycophenolate mofetil
ITP: Immune thrombocytopenic purpura
